# The Burden of Cardiovascular Disease Attributable to Hypertension in Nigeria: A *Modelling Study Using Summary-Level Data*

**DOI:** 10.5334/gh.1332

**Published:** 2024-06-06

**Authors:** Adedayo E. Ojo, Dike B. Ojji, Diederick E. Grobbee, Mark D. Huffman, Sanne A. E. Peters

**Affiliations:** 1 Julius Global Health, Julius Center for Health Sciences and Primary Care, University Medical Center, Utrecht University, 3584 CG Utrecht, The Netherlands; 2 Cardiovascular Research Unit, University of Abuja and University of Abuja Teaching Hospital, Gwagwalada, Abuja 902101, Nigeria; 3 Department of Medicine, Faculty of Clinical Sciences, University of Abuja, Gwagwalada, Abuja 902101, Nigeria; 4 Cardiovascular Division and Global Health Center, Washington University, St. Louis, MO 63110, USA; 5 The George Institute for Global Health, University of New South Wales, Sydney, NSW 2052, Australia; 6 Department of Preventive Medicine, Feinberg School of Medicine, Northwestern University, Chicago, IL 60611, USA; 7 The George Institute for Global Health, School of Public Health, Imperial College London, London, UK

**Keywords:** Hypertension, stroke, cardiovascular disease, prevalence, population attributable fraction, Nigeria

## Abstract

**Background:**

Globally, cardiovascular disease (CVD) remains the leading cause of mortality and disability, with hypertension being the single most important modifiable risk factor. Hypertension is responsible for about 18% of global deaths from CVD, of which African regions are disproportionately affected, especially sub-Saharan Africa. This study assessed the burden of major CVD subtypes attributable to hypertension in Nigeria.

**Methods:**

The population attributable fractions (PAF) for myocardial infarction, all strokes, ischaemic stroke and intracerebral haemorrhagic stroke attributable to hypertension in Nigeria were calculated using published results from the INTERHEART and INTERSTROKE studies and prevalence estimates of hypertension in Nigeria. PAF estimates were obtained for age, sex, and geopolitical zones.

**Results:**

Overall, hypertension contributed to 13.2% of all myocardial infarctions and 24.6% of all strokes, including 21.6% of all ischaemic strokes and 33.1% of all intracerebral haemorrhagic strokes. Among men aged ≤55 years, the PAF for myocardial infarction ranged from 11.7% (North-West) to 14.6% (South-East), while in older men, it spanned 9.2% (North-West) to 11.9% (South-East). Among women aged ≤65 years, PAF varied from 18.6% (South-South) to 20.8% (South-East and North-Central), and among women aged >65 years, it ranged from 10.4% (South-South) to 12.7% (South-East).

**Conclusion:**

Hypertension is a key contributor to the burden of CVD in Nigeria. Understanding the burden of hypertension in the Nigerian population overall and key subgroups is crucial to developing and implementing contextualised health policies to reduce the burden of CVD. Public health interventions and policies centred on hypertension will play a critical role in potentially alleviating the burden of cardiovascular diseases (CVD) in Nigeria.

## Introduction

Globally, cardiovascular disease (CVD) remains the leading cause of death, accounting for 32% of all global deaths [1]. Hypertension is the key modifiable risk factor for CVD. An estimated 1.3 billion people around the world have hypertension, with two-thirds of these persons living in low- and middle-income countries (LMICs) [2]. In Sub-Saharan Africa (SSA), hypertension is a major public health problem, with approximately more than 75 million people living with hypertension [3]. The burden of hypertension and consequent morbidity and mortality is expected to continue to increase, in part due to weak health systems and the poor availability of effective preventive strategies [4].

Nigeria, like most other SSA countries, is currently experiencing an epidemiological transition characterised by increasing urbanisation and changes in lifestyle factors, resulting in an increased burden of CVD [5,6]. The WHO Global Action Plan on the prevention and control of non-communicable diseases (NCDs) as well as the National Multi-sectoral Action Plan for NCDs in Nigeria both put major emphasis on the burden of hypertension and CVD [7,8,9,10]. Understanding the burden of hypertension in the Nigerian population is urgently needed to raise awareness and develop policies to reduce the burden of hypertension-related CVD in Nigeria.

In this study, we aimed to obtain a representative estimate of the burden of CVDs attributable to hypertension in Nigeria overall and in key subgroups to generate data that will drive policies on preventing CVD in Nigeria [11].

## Methods

### Study Design

This was a modelling study using summary-level data. Employing this approach from systematic reviews and global studies like INTERHEART and INTERSTROKE, the study estimated the population attributable risk of hypertension across Nigeria’s geopolitical zones. The design, integrating retrospective analysis and modelling, overcomes traditional observational study limitations, providing comprehensive insights into CVD attributable to hypertension. Modelling studies efficiently leverage existing data to assess population-level impacts, which is crucial for Nigeria’s diverse regions. The approach accommodates temporal trends, estimating the population attributable fraction for 1995 and 2020 and offering a robust, resource-efficient method to understand hypertension’s evolving impact on cardiovascular health in Nigeria.

### Study Setting

**Figure 1:**
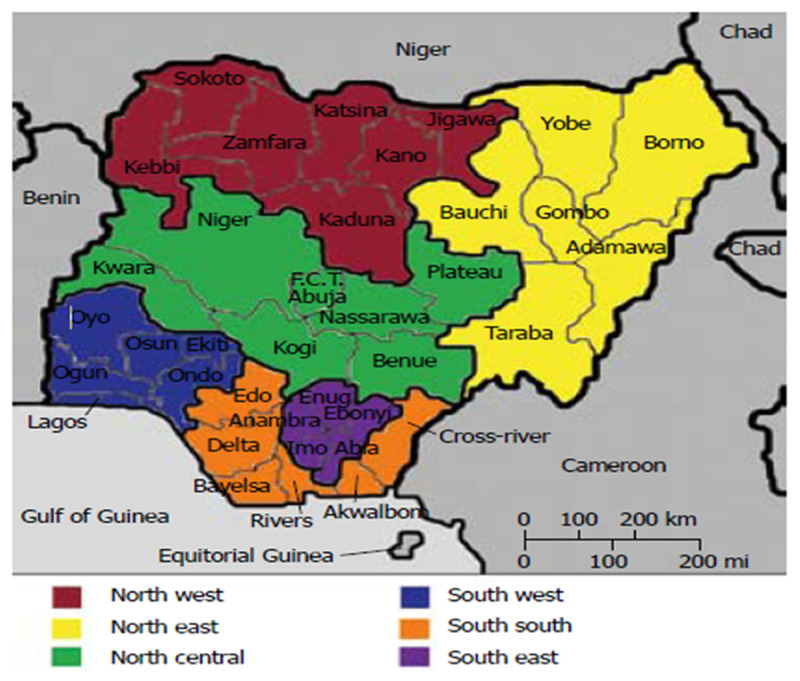
Map of Nigeria showing the six geopolitical zones, 36 states, and Federal Capital Territory [6].

Nigeria is located on the western coast of Africa [12]. With around 217 million inhabitants, it is the most populous country in Africa and the most populous Black nation on earth. The country has 36 states and its Federal Capital Territory, Abuja. The 36 states are grouped into six geopolitical zones: the North Central, North East, North West, South West, South East and South South (Figure 1) [13].

### Prevalence of Hypertension

We obtained the most recent prevalence estimates of hypertension across the six geopolitical zones in Nigeria from a previous systematic review with meta-analyses [14]. In the review, hypertension was defined as an SBP of 140 mmHg or more and a DBP of 90 mmHg or more, a self-reported history of hypertension. The review included 52 studies from the six geopolitical zones in Nigeria, including 21 studies each from urban and rural settings and 13 studies from mixed settings (rural-urban), totaling 78,949 individuals. The prevalence of hypertension was estimated for 1995 and 2020, by age and by sex.

### Risk of CHD and Stroke Associated with Hypertension

The odds ratios (OR) for myocardial infarction, overall stroke, ischaemic stroke, and intracerebral haemorrhagic stroke associated with hypertension in both men and women were obtained from INTERHEART and INTERSTROKE studies [15,16]. INTERHEART was a case-control study that included 15,152 cases with acute myocardial infarction and 14,820 controls from across 262 centres in 52 regions worldwide; a total of 578 cases and 789 controls were from Africa [17]. INTERSTROKE was a case-control study that included 663 and 2237 cases of intracerebral haemorrhagic stroke and ischaemic stroke, respectively, and 3,000 controls from 84 centres in 22 countries. A total of 141 and 214 cases of intracerebral hemorrhagic stroke and ischemic stroke were respectively reported for Africa (Nigeria, South Africa, Sudan, and Uganda were among the African countries included in the study). A self-reported history of hypertension was used to define hypertension in both studies.

### Statistical Analysis

The population attributable fraction (PAF%) was estimated using the Levin formula shown below [18]:


1 \[PAF\left( \% \right) = \,\frac{{{P_e}\ (RR - 1)}}{{[{P_e}\ \left( {RR - 1} \right) + 1]}} \times 100\%\]

Where; P_e_ is the probability of exposure to the risk factor, that is, the prevalence of hypertension in Nigeria and RR is the relative risk. Estimation of relative risks from the ORs was done using the equation below, and subsequently, the PAF for the burden of CVDs in Nigeria was obtained.


2 \[
RR = \,\frac{{OR}}{{\left( {1 - {P_e}} \right) + ({P_e}*OR)}}
\]

We estimated the above PAF% for the burden of CVDs overall and across subgroups defined by age, sex, and geopolitical zone. Sex-specific PAFs% were calculated for all the outcomes. For myocardial infarction, age- and region-specific PAFs% were computed based on the information available [14]. PAF% for all the outcomes for both 1995 and 2020 were also computed. A student t-test was used to evaluate the significance of the differences in PAF across the region. The test was conducted at a two-sided 5% level of significance with no adjustment for multiple testing. Statistical analysis was carried out using R version 4.1.0 (2021-05-18).

## Results

**Figure 2:**
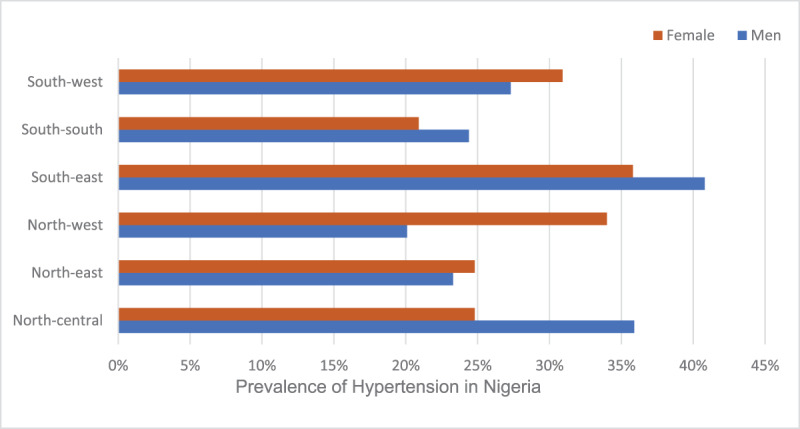
Prevalence of hypertension in the six geopolitical zones in Nigeria [14].

The reported prevalence of hypertension in Nigeria was 30.9% and ranged across the regions from 24.7% (North West) to 33.3% (South East). Men in the South East had the highest pooled prevalence of hypertension (40.8%) compared to women (35.1%), and this was also among women in the South East. The lowest prevalence of hypertension was in the North West for men (20.1%) and in the South South for women (20.9%) (Figure 2).

### Sex-specific PAFs for all outcomes

Table 1 shows the sex-specific PAF of myocardial infarction, all stroke, ischaemic stroke and intracerebral haemorrhagic stroke in the six geopolitical zones of Nigeria. Overall, 13.2% of the burden of myocardial infarction and 24.6% of the burden of all strokes could be attributed to hypertension, with slight variation between the regions. Hypertension was estimated to have contributed about 21.6% and 33.1% of the burden of ischaemic stroke and intracerebral haemorrhagic stroke, respectively.

**Table 1:** Sex-Specific PAF (%) of myocardial infarction, all stroke, ischaemic stroke and intracerebral haemorrgagic stroke in the six geopolitical zones in Nigeria.


GEOPOLITICAL ZONES	BOTH SEXES	MEN	WOMEN		

	PAF (%)	PAF (%)	PAF (%)	ABSOLUTE DIFFERENCE IN PAF%	P-VALUE

**MYOCARDIAL INFARCTION**					

North Central	13.3	13.6	12.2	1.5	0.78

North East	12.1	11.8	12.2	0.3	

North West	13.3	11.0	13.5	2.5	

South East	13.4	13.81	13.6	0.2	

South South	12.7	12.1	11.2	0.9	

South West	13.1	12.6	13.2	0.5	

Overall	13.2				

**ALL STROKE**					

North Central	24.6	24.6	23.9	0.7	0.56

North East	23.9	23.6	23.9	0.3	

North West	24.6	22.7	24.7	2.0	

South East	24.7	24.3	24.6	0.4	

South South	24.3	23.8	23.0	0.8	

South West	24.5	24.3	24.6	0.3	

Overall	24.6				

**ISCHAEMIC STROKE**					

North Central	21.7	21.8	20.7	1.1	0.66

North East	20.7	20.3	20.7	0.4	

North West	21.7	19.4	21.8	2.4	

South East	21.7	21.6	21.8	0.2	

South South	21.2	20.6	19.6	1.0	

South West	21.5	21.1	21.6	0.4	

Overall	21.6				

**INTRACEREBRAL HAEMORRGAGIC STROKE**					

North Central	33.0	32.4	33.5	1.2	0.63

North East	33.5	33.5	33.5	0.0	

North West	33.0	33.2	32.7	0.5	

South East	32.8	31.3	32.4	1.1	

South South	33.4	33.5	33.3	0.2	

South West	33.2	33.4	33.1	0.3	

Overall	33.1				


### Age-and region-specific PAF of myocardial infarction

Across regions, hypertension conferred 15.0–16.3% of the burden of myocardial infarction among younger individuals and 15.1–19.3% of the burden of myocardial infarction among older individuals. In men aged ≤55 years, the PAF for myocardial infarction ranged from 11.7% (North West) to 14.6% (South East). Among men aged >55 years, the range of PAF was 9.2% (North West) to 11.9% (South East). In women aged ≤65 years, the PAF ranged from 18.6% (South South) to 20.8% (South East and North Central). Among women aged >65 years, the PAF ranged from 10.4% (South South) to 12.7% (South East) (Table 2).

**Table 2:** Age and sex-specific PAF% of hypertension for the burden of myocardial Infarction.


REGION	BOTH SEXES			MEN			WOMEN		
		
	Young	Old	Absolute Difference	≤55 years	>55 years	Absolute Difference	≤65 years	>65 years	Absolute Difference

North Central	16.2	18.8	2.6	14.4	11.6	2.8	19.6	11.3	8.3

North East	15.0	15.1	0.1	12.6	9.9	2.7	19.6	11.3	8.3

North West	16.2	18.7	2.5	11.7	9.2	2.5	20.8	12.6	8.2

South East	16.3	19.3	3.0	14.6	11.9	2.7	20.8	12.7	8.1

South South	15.6	16.6	1.0	12.8	10.2	2.6	18.6	10.4	8.2

South West	16.0	17.9	1.9	13.4	10.7	2.7	20.6	12.3	8.3

Urban	16.3	19.5	3.2	13.4	10.7	2.7	20.8	12.6	8.2

Rural	15.2	15.5	0.3	13.2	10.5	2.7	19.2	10.9	8.3

Mixed	16.4	19.5	3.1	14.4	11.6	2.8	20.8	12.6	8.3

p-value			0.005			<0.0001			<0.0001


### Age-specific PAF of all outcomes in 1995 and 2020

**Figure 3:**
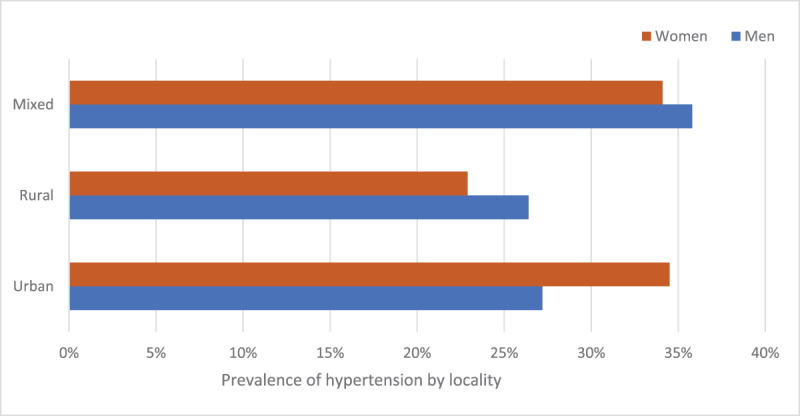
Prevalence of hypertension in the rural, urban and mixed settings in Nigeria [14].

**Figure 4:**
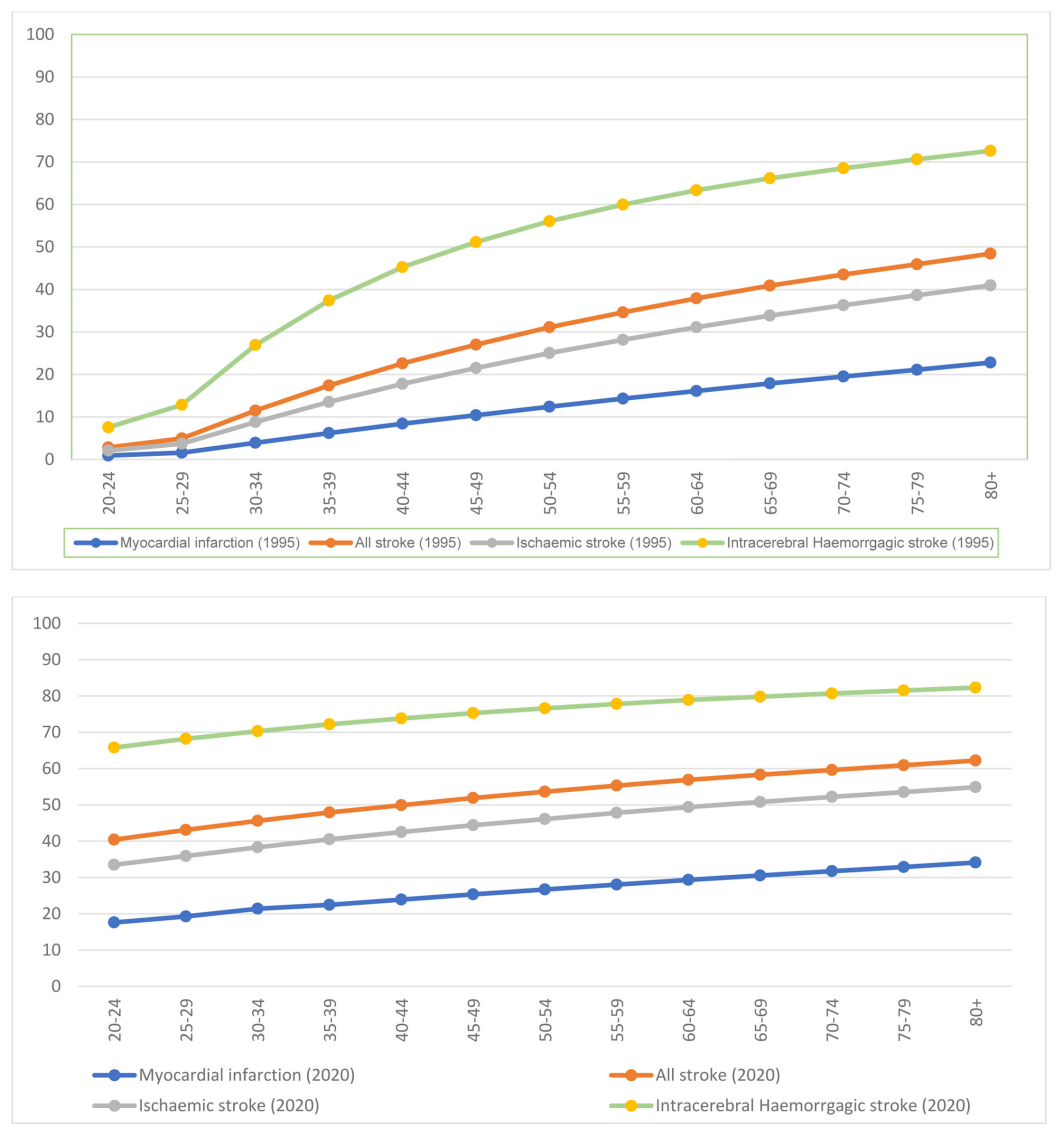
Age-specific population attributable risk of hypertension for the burden of myocardial infarction, all strokes and major stroke types in 1995 (top) and 2020 (bottom).

For myocardial infarction, the PAF increased from 6.2% in 1995 to 13.3% in 2020. For all strokes, the PAF increased from 15.4% in 1995 to 24.6% in 2020. For ischaemic stroke, the PAF increased from 12.4% in 1995 to 21.7% in 2020. For intracerebral haemorrhagic stroke, the PAF increased from 27.4% in 1995 to 32.9% in 2020. The observed differences in PAF values were found to be statistically significant, as evidenced by the p-values of <0.0001 for the comparisons except for intracerebral haemorrhagic stroke, where p > 0.05 (Figures 3 and 4; Table 3).

**Table 3:** PAF of hypertension for the burden of myocardial infarction, all strokes and major stroke types (age ≥20 years), during 1995 and 2020.


	MYOCARDIAL INFARCTION			ALL STROKE			ISCHAEMIC STROKE			INTRACEREBRAL HAEMORRGAGIC STROKE		
			
	1995	2020		1995	2020		1995	2020		1995	2020	
							
AGE CATEGORY	PAF (%)	PAF(%)	ABSOLUTE DIFFERENCE	PAF(%)	PAF(%)	ABSOLUTE DIFFERENCE	PAF(%)	PAF (%)	ABSOLUTE DIFFERENCE	PAF(%)	PAF (%)	ABSOLUTE DIFFERENCE

20–24	7.0	11.9	16.7	16.8	23.6	6.8	13.7	20.4	6.7	28.8	33.5	4.7

25–29	10.3	12.4	17.7	21.9	24.1	2.2	18.6	21.0	2.4	32.8	33.5	0.7

30–34	13.8	12.9	17.5	23.7	24.5	0.8	21.2	21.4	0.2	30.2	33.3	3.1

35–39	9.7	13.3	16.2	15.5	24.6	9.1	14.1	21.6	7.5	18.8	33.0	14.2

40–44	7.0	13.5	15.5	16.9	24.6	7.7	13.8	21.8	8.0	28.9	32.6	3.7

45–49	8.3	13.7	14.9	19.1	24.5	5.4	15.8	21.8	6.0	30.8	32.1	1.2

50–54	9.5	13.8	14.3	20.8	24.3	3.5	17.4	21.7	4.3	32.1	31.5	0.6

55–59	10.4	13.8	13.7	22.0	24.0	2.0	18.7	21.5	2.8	32.9	30.8	2.1

60–64	11.3	13.8	13.2	23.0	23.6	0.6	19.7	21.2	1.5	33.3	30.0	3.3

65–69	12.0	13.6	12.6	23.7	23.1	0.6	20.5	20.8	0.3	33.5	29.2	4.3

70–74	12.5	13.4	12.2	24.2	22.6	1.6	21.0	20.3	0.7	33.5	28.3	5.2

75–79	13.0	13.2	11.8	24.5	21.9	2.6	21.4	19.8	1.6	33.3	27.4	5.9

80+	13.3	12.8	11.3	24.6	21.1	3.5	21.7	19.1	2.6	32.9	26.2	6.7

All (20+)	6.2	13.3	15.5	15.4	24.6	9.2	12.4	21.7	9.3	27.4	32.9	5.5

P-value			0.0001			0.008			0.002			0.777


## Discussion

This study assessed the impact of hypertension on the prevalence of CVDs in Nigeria. The findings highlight the escalating burden of hypertension, which has emerged as the primary driver of CVDs. Hypertension plays a significant role in cardiovascular health, contributing to 13.2% of myocardial infarction burden and 24.6% of all stroke cases. It is associated with around 21.6% of ischaemic stroke cases, while having an even greater influence, at 33.1%, on the burden of intracerebral haemorrhagic strokes. The substantial increase in the PAF for myocardial infarction from 6.2% in 1995 to 13.3% in 2020 indicates a significant shift in the contribution of hypertension to this specific condition over time. Similarly, the escalating PAF values for all stroke cases, rising from 12.4% to 21.7%, suggest a growing impact of hypertension on the overall stroke burden. Of notable significance is the trend observed in ischaemic stroke, where the PAF surged from 15.4% to 24.6% between 1995 and 2020. This highlights a substantial increase in the proportion of ischaemic stroke cases attributed to hypertension. Additionally, the remarkable rise in the PAF for intracerebral haemorrhagic stroke, soaring from 27.4% to 32.9%, underscores a significant augmentation in the proportion of cases linked to hypertension for this specific stroke subtype. These findings collectively underscore the evolving impact of hypertension as a key contributor to the burden of myocardial infarction and various stroke types.

The observed variations in PAFs of hypertension across the geopolitical zones in Nigeria have notable implications. The South East zone displayed the most significant relative difference in the PAF for myocardial infarction at 13.4%, highlighting the potential influence of regional factors on cardiovascular health. Likewise, the PAF values for all strokes, ischaemic stroke and intracerebral haemorrhagic stroke were notably high in the South East (24.7%), ‘North Central, North West, South East, South South, South West’ (21.7% each), North West (33.4%), and North East (33.5%). These findings emphasise the varying impact of hypertension on different types of strokes and cardiovascular events in different geographical regions of Nigeria, suggesting the need for tailored interventions and strategies to address the disparities and mitigate the associated health risks. This finding aligns with previous research, as hypertension has been consistently identified as one of the most prevalent risk factors for CVDs and plays a substantial role in the escalating burden of these diseases [3,19,20]. We observed significant variations in the burden of CVDs based on age and gender.

Notably, the PAF estimates differed among various age groups, with higher attributable fractions seen in older age groups compared to younger ones, given the increasing prevalence of hypertension with age. These findings align with earlier studies in Africa, where hypertension has consistently been ranked as the major risk factor for stroke events, particularly in relation to age. For instance, in South Africa, hypertension was identified as a major contributor to the burden of stroke, with a higher PAF reported compared to Nigeria (38.0% vs. 24.6%) [21]. In a study carried out in the Ashanti Region of Ghana, lower attributable burdens of both myocardial infarction (8.8% vs. 13.2%) and stroke (17.3% vs. 24.6%) related to hypertension were observed in Ghana compared to Nigeria.

The results of this study emphasise the significant variation in the burden of CVDs attributable to hypertension across different age and sex groups. Hypertension contributes more significantly to CVDs in men, particularly in older age groups. However, implementing population-wide measures and controls would be equally beneficial for both younger and older populations, as well as for both men and women. The study suggests that hypertension, as a prevalent risk factor, may contribute significantly to the burden of CVDs across Nigeria’s six geopolitical zones. This observation underscores the importance of global efforts to prioritise the control and prevention of elevated blood pressure. This aligns with recent initiatives by the Nigerian Federal Ministry of Health, focusing on measures outlined in the National Multi-sectoral Action Plan to combat the growing cardiovascular epidemic and implementing WHO-recommended interventions (“best buys”) for the prevention and control of noncommunicable diseases, including CVD. The findings support the notion that a significant burden of cardiovascular morbidity and mortality in the Nigerian population can be preventable through timely identification and prioritisation of public health interventions, including effective population-based prevention strategies centred on CVDs in resource-constrained settings, such as population-wide sodium-reduction strategies.

Aligned with Nigeria’s National Multi-Sectoral Action Plan for the Prevention and Control of Noncommunicable Diseases (NCDs), the country is experiencing an epidemiological transition, leading to a dual burden of NCDs. Our study corroborates this trend, revealing a notable increase in the burden of CVD between 1995 and 2020. As seen in other nations, hypertension remains a dominant risk factor driving the surge in CVDs in Nigeria. However, it is crucial to emphasise that CVDs attributable to hypertension are largely preventable, and timely interventions can significantly improve outcomes. Our findings are in line with previous research in other LMICs, which also highlighted the rising prevalence of hypertension as a key driver of the increasing burden of CVDs in the regions [22,23,24]. For instance, both our study and the SIREN Stroke study showed the significant impact of hypertension on the occurrence of stroke. In our study, we observed that hypertension plays a crucial role in stroke incidence, with a PAF of 24.6% compared to 88.7% in the SIREN Stroke study. Furthermore, the SIREN Stroke study found a higher PAF for ischaemic strokes (77.5%) compared to haemorrhagic strokes (52.5%). In contrast, our study found a lower PAF for ischaemic strokes (21.6%) and a higher PAF for haemorrhagic strokes (33.1%). These discrepancies may reflect differences in the study populations, methodologies or other factors influencing the prevalence and impact of hypertension on stroke risk. Most notably, the SIREN Stroke study was conducted among young West Africans, and the odds ratio of stroke associated with hypertension was considerably higher than in INTERSTROKE. Overall, these findings emphasise the complex relationship between hypertension and stroke risk, highlighting the need for tailored prevention and management strategies based on population-specific characteristics and risk profiles [25].

This study has several strengths, including its extensive nationwide scope, covering all six geopolitical zones in Nigeria and ensuring comprehensive coverage. Additionally, the inclusion of studies with large population sizes enhances the statistical power and reliability of our findings. However, it is essential to acknowledge potential limitations in our study. One limitation of the modelling study design employed in this research is its reliance on existing data. The accuracy and applicability of the estimates are contingent upon the quality and representativeness of the data sources. Despite employing standardised data collection approaches for both cases and controls, inherent biases such as selection and recall bias in a case-control study could have influenced the results. However, these biases are likely to have minimal impact on the observed time trends. Another limitation is the inability to assess the PAF for fatal myocardial infarction or stroke, as participants in the INTERHEART and INTERSTROKE studies were those who survived their initial event.

## Conclusion

The population-attributable risk fractions for various CVDs in Nigeria were found to be substantial. Notably, the burden of hypertension-attributable CVDs has significantly increased from 1995 to 2020, with notable differences observed across age groups, with older individuals bearing the highest burden. Hypertension has been identified as a primary driver of CVDs in Nigeria, presenting a significant opportunity for prevention. Public health interventions and policies focusing on hypertension are crucial to potentially reducing the burden of CVD in the country. Building upon existing interventions like the National Multi-Sectoral Action Plan, it is essential to implement additional population-based prevention strategies to address the control of CVDs effectively. In line with WHO recommendations, enhancing health promotion interventions and awareness campaigns regarding hypertension risk factors becomes imperative, with the goal of reducing hypertension prevalence by 30%.

## Appendix

**Table S3:** Sex-specific PAF (%) of myocardial infarction, all stroke, ischaemic stroke and intracerebral haemorrgagic stroke by level of rurality.

LEVEL OF RURALITY	BOTH SEXES	MEN	WOMEN	ABSOLUTE DIFFERENCEIN PAF%	P-VALUE

**MYOCARDIAL INFARCTION**	PAF (%)	PAF (%)	PAF (%)		

Urban	13.5	12.6	13.5	0.9	0.999

Rural	12.3	12.5	11.7	0.7	

Mixed	13.5	13.6	13.5	0.1	

**ALL STROKE**					

Urban	24.7	24.3	24.6	0.4	0.816

Rural	24.0	24.2	23.5	0.67	

Mixed	24.7	24.6	24.7	0.04	

**ISCHAEMIC STROKE**					

Urban	21.7	21.1	21.8	0.6	0.958

Rural	20.8	21.0	20.2	0.8	

Mixed	21.7	21.8	21.8	0.0	

**INTRACEREBRAL HAEMORRHAGIC STROKE**					

Urban	32.7	33.4	32.6	0.8	0.731

Rural	33.5	33.5	33.5	0.0	

Mixed	32.7	32.4	32.7	0.3	

## References

[1] Lafeber M, Spiering W, Visseren FLJ, Grobbee DE. Multifactorial prevention of cardiovascular disease in patients with hypertension: the cardiovascular polypill. Current Hypertension Reports. 2016; 18(5): 1–7. DOI: 10.1007/s11906-016-0648-3 27083928 PMC4833798

[2] World Health Organization. Hypertension Indicators for Improving Quality and Coverage of Services; 2021.

[3] Guwatudde D, Nankya-Mutyoba J, Kalyesubula R, Laurence C, Adebamowo C, Ajayi I, Bajunirwe F, Njelekela M, Chiwanga FS, Reid T, et al. The burden of hypertension in sub-Saharan Africa : A four-country cross sectional study. BMC Public Health. 2015; 15: 1–8. DOI: 10.1186/s12889-015-2546-z 26637309 PMC4670543

[4] Cappuccio FP, Miller MA. Cardiovascular disease and hypertension in sub-Saharan Africa: Burden, risk and interventions. Internal and Emergency Medicine. 2016; 11(3): 299–305. DOI: 10.1007/s11739-016-1423-9 27001886 PMC4820479

[5] Oyeyemi AL, Adeyemi O. Relationship of physical activity to cardiovascular risk factors in an urban population of Nigerian adults. Archives of Public Health. 2013; 71(1): 1–9. DOI: 10.1186/0778-7367-71-6 23578186 PMC3635946

[6] Ogah OS, Okpechi I, Chukwuonye II, Akinyemi JO, Onwubere BJ, Falase AO, Stewart S, Sliwa K. Blood pressure, prevalence of hypertension and hypertension related complications in Nigerian Africans: A review. World Journal of Cardiology. 2012; 4(12): 327–340. DOI: 10.4330/wjc.v4.i12.327 23272273 PMC3530788

[7] Abdullahi A. National Multi-Sectorial Action Plan for the Non-Communicable Diseases. Federal Ministry of Health, 2019.

[8] Roth GA, Johnson C, Abajobir A, Abd-Allah F, Abera SF, Abyu G, Ahmed M, Aksut B, Alam T, Alam K, et al. Global, regional, and national burden of cardiovascular diseases for 10 causes, 1990 to 2015. Journal of the American College of Cardiology. 2017; 70(1): 1–25. DOI: 10.1016/j.jacc.2017.04.052 28527533 PMC5491406

[9] WHO. ‘Best Buys’ and Other Recommended Interventions for the Prevention and Control of Noncommunicable Diseases; the updated Appendix 3 of the WHO Global NCD Action Plan 2013-2020. WHO Glob. NCD Action Plan 2013-2020 2017. 2017; 65–70. https://www.who.int/publications/i/item/WHO-NMH-NVI-17.9.

[10] World Health Organization. Action plan for the prevention and control of noncommunicable diseases in the WHO European Region 2016–2025; 2016.

[11] Boateng D, Wekesah F, Browne JL, Agyemang C, Agyei-Baffour P, De-Graft Aikins A, Smit HA, Grobbee DE, Klipstein-Grobusch K. Knowledge and awareness of and perception towards cardiovascular disease risk in sub-Saharan Africa: A systematic review. PLoS One. 2017; 12(12): 1–21. DOI: 10.1371/journal.pone.0189264 PMC5726714 29232703

[12] African Development Bank Group. Tracking Africa’s Progress in Figures; 2014.

[13] Okorie PN, Ademowo GO, Saka Y, Davies E, Okoronkwo C. Lymphatic filariasis in Nigeria ; Micro-stratification overlap mapping (MOM) as a prerequisite for cost-effective resource utilization in control and surveillance. PLoS Neglected Tropical Diseases. 2013; 7(9): e2416. DOI: 10.1371/journal.pntd.0002416 24040432 PMC3764235

[14] Adeloye D, Owolabi EO, Ojji DB, Auta A, Dewan MT, Olanrewaju TO, Ogah OS, Omoyele C, Ezeigwe N, Mpazanje RG, et al. Prevalence, awareness, treatment, and control of hypertension in Nigeria in 1995 and 2020: A systematic analysis of current evidence. Journal of Clinical Hypertension. 2021; 23(5): 963–977. DOI: 10.1111/jch.14220 33600078 PMC8678849

[15] Donnell MJO, Xavier D, Liu L, Zhang H, Chin SL, Rao-Melacini P, Rangarajan S, Islam S, Ardila SC, Foscal L. Risk factors for ischaemic and intracerebral haemorrhagic stroke in 22 countries (the INTERSTROKE study): A case-control study. Lancet. 2010; 376(9735): 112–123. DOI: 10.1016/S0140-6736(10)60834-3 20561675

[16] Mcqueen MJ, Hawken S, Wang X, Ounpuu S, Sniderman A, Steyn K, Sanderson JE. Lipids, lipoproteins, and apolipoproteins as risk markers of myocardial infarction in 52 countries (the INTERHEART study): A case-control study. Lancet. 2008; 372(9634): 224–233. DOI: 10.1016/S0140-6736(08)61076-4 18640459

[17] Yusuf S, Hawken S, Ôunpuu S, Dans T, Avezum A, Lanas F, Mcqueen M, Budaj A, Pais P. Effect of potentially modifiable risk factors associated with myocardial infarction in 52 countries (the INTERHEART study): Case-control study. Lancet. 2004; 364(9438): 937–952. DOI: 10.1016/S0140-6736(04)17018-9 15364185

[18] Hussain MA, Mamun AA, Peters SAE, Woodward M, Huxley RR. The burden of cardiovascular disease attributable to major modifiable risk factors in Indonesia. Journal of Epidemiology. 2016; 26(10): 515–521. DOI: 10.2188/jea.JE20150178 27021286 PMC5037248

[19] Hendriks ME, Wit FWNM, Roos MTL, Brewster LM, Akande TM, de Beer IH, Mfinanga SG, Kahwa AM, Gatongi P. Hypertension in sub-Saharan Africa: Cross-sectional surveys in four rural and urban communities. PLoS One. 2012; 7(3): e32638. DOI: 10.1371/journal.pone.0032638 22427857 PMC3299675

[20] Mills KT, Stefanescu A, He J. The global epidemiology of hypertension. Nature Reviews: Nephrology. 2020; 16(4): 223–237. DOI: 10.1038/s41581-019-0244-2 32024986 PMC7998524

[21] Maredza M, Bertram MY, Gómez-Olivé, XF, Tollman SM. Burden of stroke attributable to selected lifestyle risk factors in rural South Africa. BMC Public Health. 2016; 16. DOI: 10.1186/s12889-016-2805-7 26869067 PMC4751665

[22] Owolabi MO, Sarfo F, Akinyemi R, Gebregziabher M, Akpa O, Akpalu A, Wahab K, Obiako R, Owolabi L, Ovbiagele B. Dominant modifiable risk factors for stroke in Ghana and Nigeria (SIREN): a case-control study. Lancet Glob Health. 2018; 6(4)e446. DOI: 10.1016/S2214-109X(18)30002-0 PMC5906101 29496511

[23] Shukuri A, Tewelde T, Shaweno T. Prevalence of old age hypertension and associated factors among older adults in rural Ethiopia. Integrated Blood Pressure Control. 2019; 12: 23–31. DOI: 10.2147/IBPC.S212821 31564965 PMC6745405

[24] Sani RN, Connelly PJ, Toft M, Rowa-Dewar N, Delles C, Gasevic D, Karaye KM. Rural-urban difference in the prevalence of hypertension in West Africa: A systematic review and meta-analysis. Journal of Human Hypertension. 2024; 38(4): 352–364. DOI: 10.1038/s41371-022-00688-8 35430612 PMC11001577

[25] Sarfo FS, Ovbiagele B, Gebregziabher M, Wahab K, Akinyemi R, Akpalu A, Akpa O, Obiako R, Owolabi L, Jenkins C, et al. Stroke among young West Africans: Evidence from the SIREN (stroke investigative research and educational network) large multisite case-control study. Stroke. 2018; 49(5): 1116–1122. DOI: 10.1161/STROKEAHA.118.020783 29618553 PMC5916042

